# Corneal Nerve Regeneration via MSC‐Derived EVs: Tissue Source and Culture Dimensionality Dictate miRNA Cargo and Therapeutic Efficacy

**DOI:** 10.1002/smll.202507451

**Published:** 2025-12-03

**Authors:** Hamed Massoumi, Eitan A. Katz, Melinda Alviar, Qiang Zhou, Mauricio Gonzalez Oyarzun, Deepshikha Tewari, Makayla Dove, Hanieh Niktinat, Tara Nguyen, Seyed Mahbod Baharnoori, Khandaker Anwar, Mark Maienschein Cline, Yuanxiang Li, Xiaowei Wang, Victor H. Guaiquil, Mark I. Rosenblatt, Ali R. Djalilian, Elmira Jalilian

**Affiliations:** ^1^ Department of Ophthalmology and Visual Sciences Illinois Eye and Ear Infirmary. University of Illinois Chicago Chicago IL 60612 USA; ^2^ Richard and Loan Hill Department of Biomedical Engineering University of Illinois Chicago Chicago IL 60607 USA; ^3^ Department of Pharmacology and Regenerative Medicine University of Illinois Chicago Chicago Illinois 606012 USA; ^4^ University of Illinois Cancer Center Chicago Illinois 60612 USA

**Keywords:** 3D culture, corneal nerves, exosomes, extracellular vesicles, mesenchymal stem cell

## Abstract

Extracellular vesicles (EVs) are emerging as critical mediators of intercellular communication and tissue repair, offering a promising cell‐free platform for regenerative therapies. In the cornea, sensory nerves are critical for maintaining epithelial integrity and ocular homeostasis. Nerve injury resulting from trauma, surgery, or disease leads to persistent epithelial defects and impaired vision, with limited treatment options. Here, the neuro‐regenerative potential of mesenchymal stem cell‐derived EVs (MSC‐EVs) isolated from human cornea (Co‐MSC) and bone marrow (BM‐MSC) cultured under 2D and 3D conditions is investigated. EVs are characterized by nanoparticle tracking analysis, ExoView profiling, and Western blot, and their effects on nerve regeneration are evaluated using primary trigeminal ganglion neurons in vitro and a murine corneal injury model in vivo. EVs from both tissue sources promoted neurite outgrowth; however, 3D‐derived EVs demonstrate superior efficacy compared to 2D‐derived EVs in vitro and in vivo. Co‐MSC‐EVs show a consistent trend toward enhanced regenerative effects over BM‐MSC‐EVs. Small RNA sequencing reveals that EV cargo is influenced by both tissue origin and culture dimensionality, with Co‐MSC‐EVs enriched in miRNAs regulating the extracellular matrix and immune pathways, while BM‐MSC‐EVs are enriched in neurotrophic signaling miRNAs. These findings support the rational design of MSC‐EV‐based therapies for neuro‐ophthalmic repair.

## Graphical abstract

Mesenchymal stem cell‐derived extracellular vesicles (EVs) from human cornea and bone marrow, cultured in 2D and 3D conditions, are characterized and tested for corneal nerve repair. 3D‐derived EVs show superior therapeutic efficacy and in vivo reinnervation. Small‐RNA sequencing reveals tissue‐ and dimensionality‐dependence for EV cargo that implicates neurotrophic and ECM‐remodeling pathway activation, guiding EV design for neuro‐ophthalmic therapy.

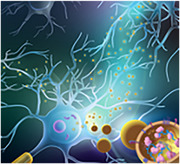

## 1 Introduction

The human cornea is recognized as the most densely innervated tissue in the body, receiving its sensory innervation from the ophthalmic (V1) branch of the trigeminal ganglion (TgV1) within the brain. Neurons originating from TgV1 project nociceptive, mechanosensitive, and cold‐sensitive fibers, which terminate on or near the corneal surface.^[^ ^1^, ^2^ ^]^ Upon entering the corneal periphery, these fibers form large nerve bundles by losing their myelin sheaths ≈1 mm past their entry point.^[^ ^1^, ^3^ ^]^ They then branch into smaller, anteriorly directed fibers that penetrate Bowman's layer, forming the initial segments of the corneal nerves. In the sub‐basal plexus, these corneal nerves create a dense neural network from which finer filaments extend through the epithelial layers to innervate the ocular surface.^[^ ^1^, ^4^, ^5^, ^6^ ^]^ Corneal nerves can be easily damaged due to their exposed location on the ocular surface and susceptibility to numerous infectious, surgical, and iatrogenic factors. Alterations in corneal nerve structure or function can give rise to a spectrum of ocular surface disturbances, ranging from reduced or absent corneal sensation (hypoesthesia or anesthesia) to persistent neuropathic pain and chronic irritation.^[^ ^7^, ^8^, ^9^, ^10^ ^]^ After injury, the regeneration of corneal sensory nerve endings proceeds slowly and is often incomplete, preventing full restoration of original nerve density and configuration. As a result, corneal sensation may remain diminished, leading to complications that include mild discomfort and, in severe cases, chronic pain.^[^ ^11^, ^12^, ^13^, ^14^ ^]^

Current therapeutic strategies for corneal nerve regeneration largely rely on single agents, including microRNAs (miRNAs) or growth factors such as nerve growth factor (NGF) and insulin growth factor (IGF).^[^ ^15^, ^16^ ^]^ However, the incomplete recovery of full nerve density highlights the need for improved therapies that may yield better anatomical and functional outcomes. Over the past decade, mesenchymal stem cell (MSC) based therapies have garnered significant attention for treating various ocular diseases.^[^ ^12^, ^17^, ^18^ ^]^ MSCs, found in adult tissues including the bone marrow (BM‐MSCs), cornea (Co‐MSCs), and adipose tissue, are central to ocular repair due to their anti‐inflammatory, anti‐fibrotic, anti‐angiogenic, and regenerative capacities.^[^ ^19^, ^20^, ^21^, ^22^, ^23^, ^24^ ^]^ Recently, increasing focus has been placed on the therapeutic potential of MSC‐derived extracellular vesicles (EVs), which are critical mediators of the molecular processes required for tissue repair and regeneration.^[^ ^4^, ^25^, ^26^, ^27^ ^]^ EVs are nanoparticles ranging from 50 to 150 nm that shuttle bioactive components (including proteins, lipids, and RNA) among cells.^[^ ^25^, ^26^, ^28^, ^29^ ^]^ For example, Co‐MSC‐derived EVs have been shown to enhance corneal wound healing and reduce scar formation by modulating inflammatory responses, promoting epithelial regeneration, and inhibiting fibroblast‐mediated scarring.^[^ ^30^, ^31^ ^]^ Similarly, EVs derived from BM‐MSCs have been implicated in both corneal wound healing and nerve regeneration, as evidenced by their ability to promote corneal epithelial repair and enhance axonal growth in cortical neurons.^[^ ^12^, ^32^, ^33^ ^]^

**Figure 1:**
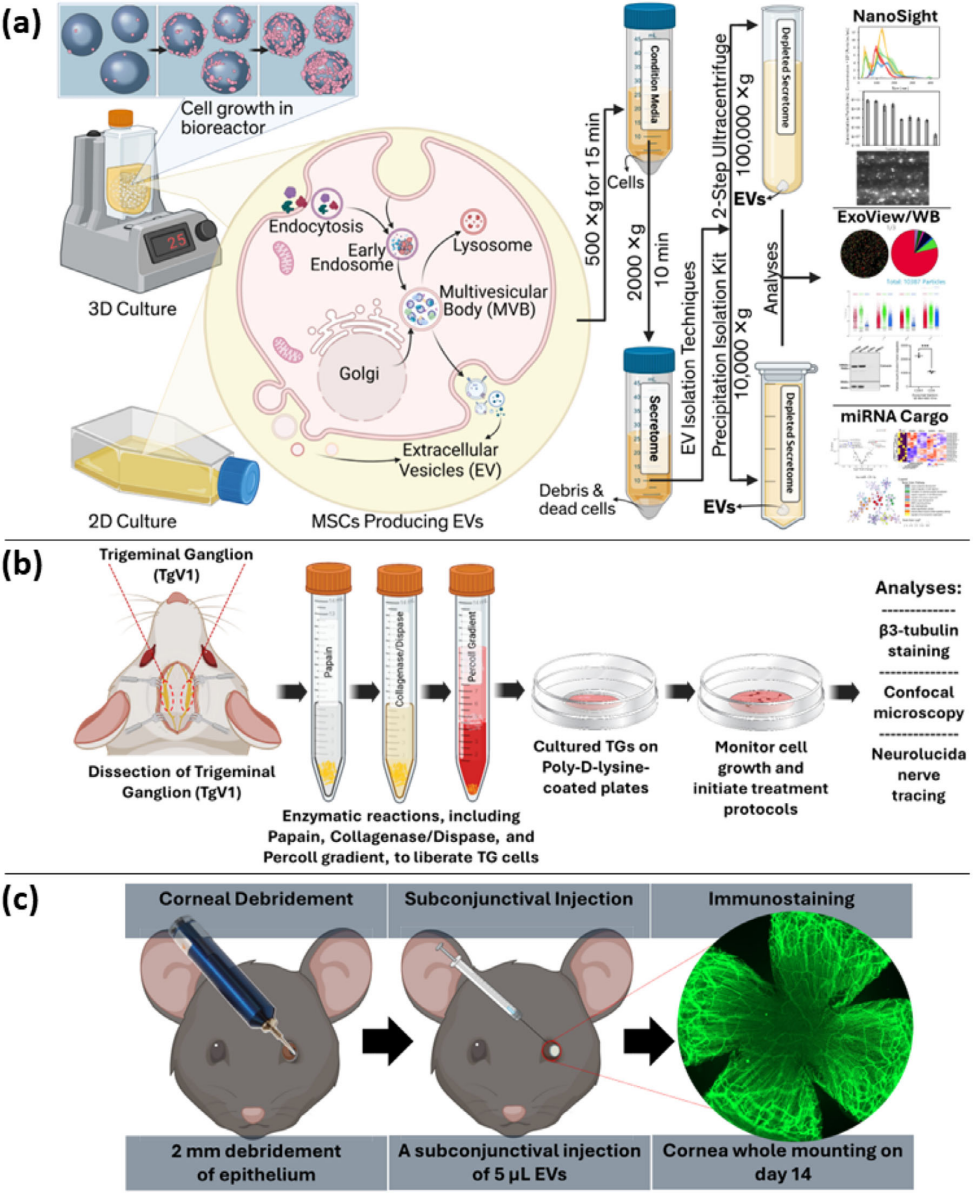
Schematic image of experimental procedure including cell culture, EV extraction, characterization, and evaluation of their regenerative efficacy in vitro and in vivo. a) MSCs derived from corneal and bone marrow sources were expanded using two culture systems: conventional flat flasks (2D culture) and 3D bioreactors (3D culture). EVs were extracted from the culture medium using ultracentrifugation or a precipitation isolation kit and characterized using NanoSight, ExoView, and Western blot platforms. b) An in vitro model was employed to assess the effects of EVs on nerve regeneration. Neurons were isolated from rodent TgV1 and treated with the extracted EVs in the culture medium. Neurite elongation was evaluated using β III tubulin staining, confocal imaging, and Neurolucida software analysis. c) An in vivo rodent corneal epithelium debridement model was used to investigate the efficacy of EVs in corneal nerve regeneration within a complex physiological environment. Following corneal injury and EV injection, corneas were dissected, stained, and imaged, and nerve regeneration was quantified using Neurolucida software. Created in BioRender. Massoumi, H. (2025) https://BioRender.com/h46mzwa.

Culture conditions, through modulation of mechanical cues, oxygen tension, and cell‐matrix interactions, play a critical role in regulating EV biogenesis, cargo composition, and functional properties. Optimizing these parameters is therefore essential not only for improving EV yield and reproducibility but also for tailoring their therapeutic efficacy for specific applications such as neuroregeneration, immunomodulation, and tissue repair.^[^ ^34^, ^35^ ^]^ In this study, we conducted a comprehensive analysis of EVs derived from Co‐MSCs and BM‐MSCs, which represent neuroectodermal and mesodermal lineages, respectively. Because conventional 2D monolayer culture may not fully recapitulate the physiological microenvironment, whereas 3D bioreactor systems better mimic in vivo conditions and have been shown to enhance EV yield and functionality,^[^ ^12^, ^36^ ^]^ we cultured these cells under both 2D and 3D conditions to systematically assess the impact of culture dimensionality. We characterized the resulting EVs using nanoparticle tracking analysis (NTA), ExoView profiling, and Western blotting. Their neuroregenerative capacity was assessed in vitro using primary trigeminal ganglion neurons (TgV1) and in vivo using a murine corneal mechanical injury model (**Figure** **1**). In addition, we performed small RNA sequencing to evaluate how tissue origin and culture dimensionality influence the molecular cargo of MSC‐derived EVs. This work provides a systematic framework to understand how biophysical culture parameters and tissue‐specific differences shape EV composition and regenerative potential, with the goal of advancing EV‐based therapies for corneal nerve repair.

## 2 Results

### 2.1 Characterization of EVs

**Figure 2:**
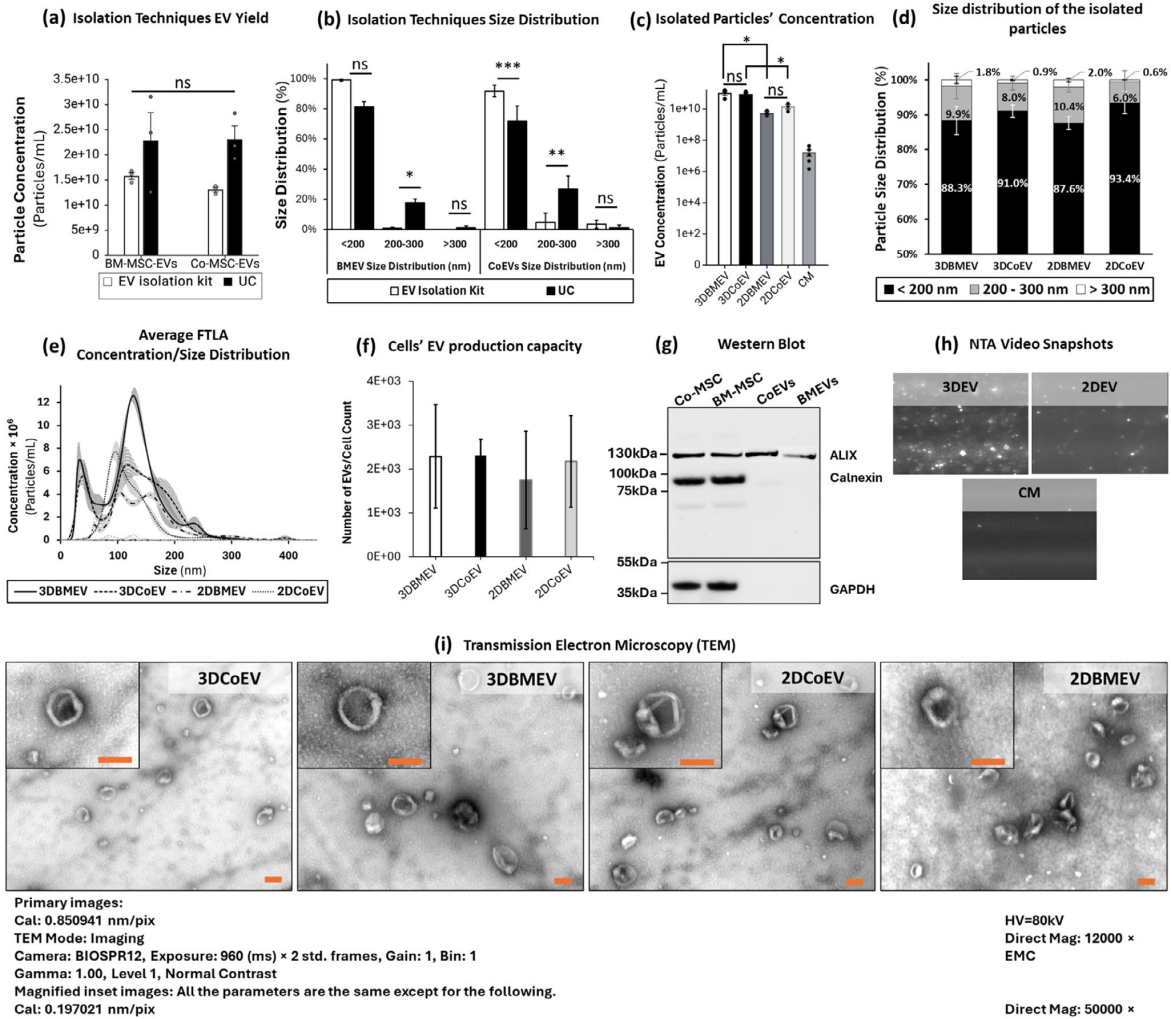
a) Between the two isolation techniques of UC and the precipitation‐based commercial kit, UC showed a higher EV yield, though the difference was not statistically significant. b) The commercial kit isolated a higher percentage of smaller EVs compared to UC, with a statistically significant difference observed in CoEVs but not in BMEVs. c) NTA analysis showed a significantly higher particle concentration in EVs derived from 3D‐cultured MSCs compared to their 2D‐cultured counterparts (p < 0.05), indicating enhanced EV yield in 3D cultures. d) Size distribution analysis revealed that over 80% of particles from all four EV groups had a diameter of less than 200 nm, consistent with the exosome size range. e) The average FTLA concentration/size distribution plot shows a similar pattern for all EV samples, with peaks below 200 nm. f) The number of EVs produced per cell was comparable across all study groups, with no significant differences observed. g) Western blot analysis confirmed the identity and purity of the EVs. EV fractions were enriched for the exosomal marker ALIX, while cellular contamination markers Calnexin and GAPDH were absent. In contrast, parent cell lysates contained all three proteins. h) Representative NTA video snapshots show a higher particle concentration in 3DEV samples compared to 2DEV samples, with minimal particles detected in the unconditioned collection media (CM). i) Transmission Electron Microscopy (TEM) images of all EV groups confirm the presence of nanoparticles with the characteristic cup‐shaped and spherical morphology consistent with exosomes. The highlights in the line graph and error bars in the bar graphs represent SE. The asterisk signs represent a significant difference (^*^ *p* < 0.05, ^**^ *p* < 0.01, ^***^ *p* < 0.001). n = 3 replicates for each condition. Scale bars = 200 nm.

EVs were isolated from the secretome of human Co‐MSCs and BM‐MSCs cultured in 2D or 3D environments using two methods: UC, which isolates EVs based on size and density through high‐speed centrifugal forces,^[^ ^37^ ^]^ and a commercial EV isolation kit (Exosome Isolation Kit, Thermo Fisher), which employs a polymer‐based precipitation method that reduces the aqueous solubility of EVs, facilitating their aggregation and subsequent recovery via low‐speed centrifugation.^[^ ^38^ ^]^ The size distribution of the isolated EVs was analyzed using NanoSight.^[^ ^39^ ^]^ Particle concentration analysis indicated that the UC technique produced a higher EV yield for both cornea‐ and bone marrow‐derived EVs, but the difference was not statistically significant (**Figure** **2**a). Overall, the commercial isolation kit preferentially isolated smaller EVs (<200 nm), with a significant enrichment observed in CoEVs but not in BMEVs (Figure 2b). Small EVs are of particular interest due to their enhanced therapeutic potential. Given that UC is a mechanically intensive method that may compromise EV integrity and functional properties, the commercial kit was chosen to ensure optimal preservation for downstream analyses.^[^ ^26^ ^]^

The average particle concentration obtained from NTA for 3D‐cultured BM‐MSCs and Co‐MSCs was (1.03 ± 2.72) × 10^11^ and (8.64 ± 1.96) × 10^10^ particles/mL, respectively, representing a statistically significant increase compared to their 2D‐cultured counterparts, which yielded (5.37 ± 1.35) × 10^9^ and (1.41 ± 1.32) × 10^10^ particles/mL respectively (p < 0.05) (Figure 2c). Size distribution analysis of EVs isolated from 3D‐ and 2D‐cultured MSCs revealed a significant enrichment of exosome‐sized particles. From 3D‐cultured MSCs, a substantial portion, 88.3% ± 4% of particles from BM‐MSCs and 91.0% ± 2% of particles from Co‐MSCs, exhibited diameters less than 200 nm, which aligns with the typical size range attributed to exosomes.^[^ ^1^, ^25^, ^26^ ^]^ The size distribution of EVs from 2D‐cultured MSCs displayed a similar trend. Here, 87.6% ± 2% and 93.4 ± 3% of particles from BM‐MSCs and Co‐MSCs, respectively, fell within the exosome size range (<200 nm) (Figure 2d). Moreover, the size distribution analysis is reflected in the average FTLA concentration/size distribution diagram with peaks below 200 nm for all the EV samples. Importantly, all EV batches, regardless of cell type or culture condition, exhibited significantly higher particle concentrations than their corresponding depleted media batches. Representative images confirmed minimal to no detectable particles in depleted media (Figure 2e). Additionally, the analysis demonstrated that all four groups (3D/2DBMEVs and 3D/2DCoEVs) produced comparable EV yields, with an average of (2.31 ± 0.5) × 10^3^ particles per cell at the time of harvest, calculated by normalizing NTA‐derived particle counts to the number of viable MSCs at harvest. Statistical evaluation revealed no significant differences among the study groups (Figure 2f). EV concentrations were quantified and normalized to the corresponding cell count to assess EV production per cell in 3D‐ and 2D‐cultured MSCs. Western blot analysis confirmed the presence of key protein markers in Co‐MSC and BM‐MSC lysates while validating the purity of the extracted EV fractions and the identity and purity of the extracted EV fractions. The samples demonstrated clear enrichment for the canonical exosomal marker ALIX, an Endosomal Sorting Complexes Required for Transport (ESCRT)‐associated protein integral to exosome biogenesis, which serves as a canonical intraluminal marker to confirm the identity of isolated EV populations.^[^ ^40^ ^]^ GAPDH was detected in whole‐cell lysates, confirming successful protein extraction. Notably, Calnexin, an endoplasmic reticulum marker, was present in cell lysates but absent in EV fractions, indicating minimal contamination of cellular or organelle.^[^ ^41^ ^]^ These findings align with MISEV 2023 guidelines,^[^ ^26^ ^]^ confirming the purity of the isolated EVs (Figure 2g). NTA video snapshots of representative samples (same dilution factor) revealed a marked increase in particle concentration in 3DEV samples compared to 2DEVs, while the CM exhibited minimal to no detectable particles (Figure 2h). To further validate the morphology of the isolated particles, TEM was performed. Representative images from all four EV groups confirmed the presence of nanoparticles exhibiting the characteristic cup‐shaped and spherical morphology consistent with exosomes, with sizes aligning with the NTA data (Figure 2i).^[^ ^42^ ^]^

These findings indicate that 3D and 2D culture methods result in the isolation of EVs enriched with exosomes, with minimal impact on the overall size distribution.

### 2.2 ExoView Analysis of EVs Demonstrated a Unique Tetraspanin Expression Profile

**Figure 3:**
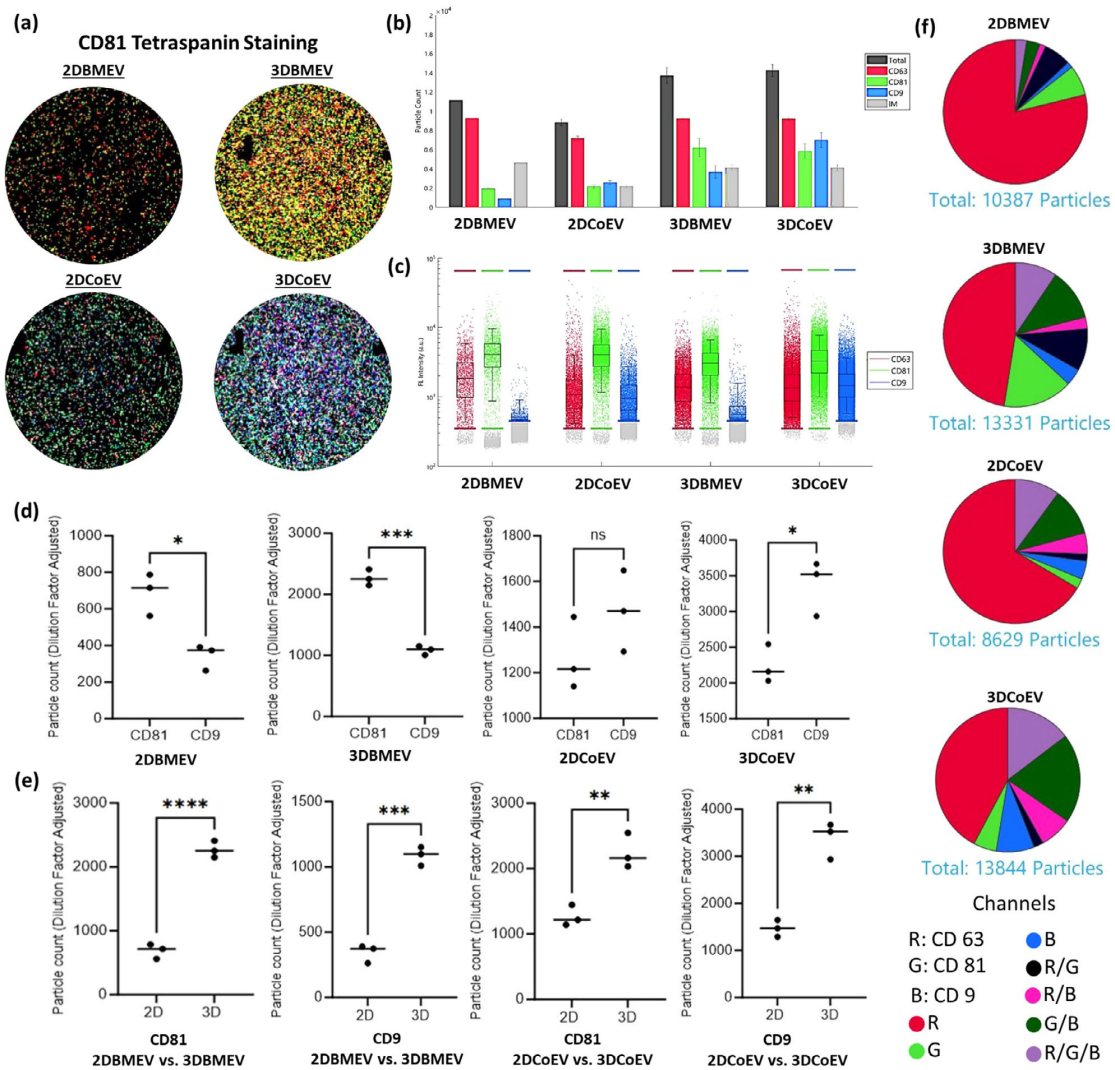
EV Characterization using ExoView. a) Tetraspanin antibody‐based capture was used to detect and identify EV subpopulations based on the expression of specific tetraspanin markers. b,c) The copy number of exosomal markers (CD63, CD81, and CD9) was quantified and presented as a bar graph for 2D‐ and 3D‐derived EVs from BM‐MSCs and Co‐MSCs, followed by fluorescence intensity analysis per EV for each marker. Isotype‐matched (IM) control antibodies were included to assess non‐specific binding. Signals obtained from IM controls were consistently low across all samples and were not used for EV quantification. Only signals above the IM threshold were considered specific. d) Statistical analysis revealed significant differences in CD81 and CD9 expression patterns within BMEVs and CoEVs. Specifically, CD81 expression was significantly higher than CD9 in both 2D and 3D BMEVs (P < 0.05), whereas CD9 expression was significantly higher than CD81 in CoEVs under both 2D and 3D conditions (P < 0.05). e) Additionally, CD81 and CD9 expression levels were significantly higher in 3DEVs compared to 2DEVs in both BM‐MSC and Co‐MSC groups. f) Representative pie charts from colocalization analysis illustrate the percentage of total EVs expressing individual tetraspanins or their combinations, highlighting the heterogeneity within each EV population. Notably, 3D cultures exhibited a greater shift toward a heterogeneous EV profile compared to 2D cultures, with CoEVs displaying higher heterogeneity than BMEVs. n = 3 biological replicates.

The characterization of 2D and 3D‐derived EVs from BM‐MSCs and Co‐MSCs using ExoView (**Figure** **3**) revealed significant differences in the expression patterns of exosomal markers. Utilizing fluorescent tetraspanin antibody staining, we could identify all the tetraspanin‐positive particles to confirm the presence of EVs (Figure 3a). Copy numbers of exosomal markers (CD63, CD81, CD9) were quantified and compared among extracted particles (Figure 3b). The marker sub‐population expression was measured in each batch to identify the expression patterns among EVs (Figure 3c). We further analyzed the statistical difference in marker expression among different samples (Figure 3d,e). Specifically, while CD81 was predominantly expressed in BMEVs, CD9 exhibited higher expression in CoEVs. Moreover, 3D culture conditions resulted in higher expression levels of both CD81 and CD9 compared to 2D conditions in both bone marrow and cornea samples. The colocalization data (Figure 3f) showed that Co‐MSCs displayed a more heterogeneous marker expression profile compared to BM‐MSCs. Additionally, 3D cultures from both cornea and bone marrow exhibited a more heterogeneous profile compared to their 2D counterparts. Furthermore, overall particle quantification demonstrated that 3DCoEVs exhibited significantly higher CD9 expression compared to 3DBMEVs as well as both 2DCoEVs and 2DBMEVs. These observations demonstrate that tissue origin and culture dimensionality significantly influence the surface marker composition and heterogeneity of MSC‐derived EVs, shaping their biogenesis and potentially affecting their regenerative function.

### 2.3 EVs Promote TgV1 Neuronal Growth In Vitro

**Figure 4:**
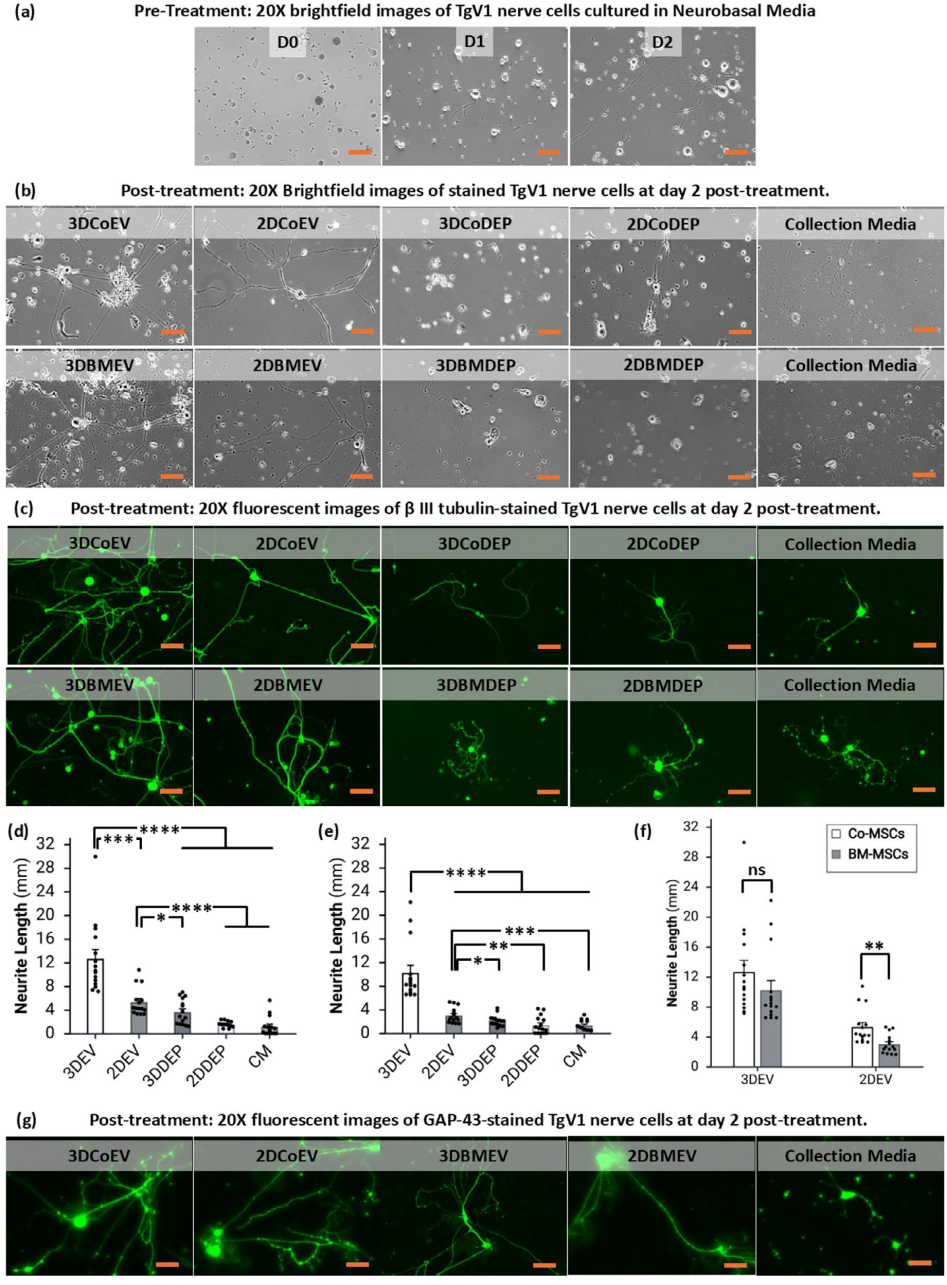
In vitro functional characterization of TgV1 neurite growth following MSC EV treatment. a) The brightfield images showed the pre‐treatment neurite growth up to day 2. b) Brightfield images with a 20× magnification were obtained from the cells after 48 h of treatment. The images showed remarkable growth in neurites treated with 3DEVs compared to 2DEV treatment and control groups. c) β III tubulin fluorescent imaging confirmed significantly enhanced nerve growth in the 3DEV‐treated group compared to the 2DEV‐treated and control groups. d–f) Quantification analysis (Neurolucida) demonstrated that neurite elongations in the presence of 3DEV treatments from BMEVs and CoEVs were significantly higher compared to their 2DEV‐treated groups. The error bars in the bar graph represent SE. The non‐significant (ns) differences between groups in the bar graph were illustrated. g) GAP‐43 immunofluorescence of TgV1 neurons illustrated an increased regeneration‐associated signal with 3DEVs relative to 2DEVs and CM control. The scale bar is 300 µm in every image. All images were taken using a 20× lens. n = 3 biological replicates for each condition. CM stands for unconditioned collection media.

The growth‐promoting effects of CoEVs and BMEVs were investigated in vitro through a primary neuronal culture. TgV1 neurons were grown in neuro‐basal media for 2–3 days until minimal neurite growth was observed in plates (**Figure** **4**a). We previously showed that the initial neurite growth is essential for the cells to be responsive to the treatments.^[^ ^12^ ^]^ Therefore, treatment processes were started on every plate on day 2 or 3 post‐culture. Cells were monitored for 2 days after treatment (Figure 4b), fixed, and stained with β III Tubulin antibody, and imaged using a fluorescent microscope (Figure 4c). Qualitative analyses of the images showed that in the 3DCoEV and 3DBMEV treatment groups, the EVs tend to create enhanced neurite elongation, thickness, and observable complexity in every plate. The morphological analysis of the images confirms that both 2D‐ and 3D‐derived EVs from Co‐MSCs and BM‐MSCs enhance TgV1 neurite growth. However, 3DEVs exhibited a significantly greater effect, indicating their superior neuroregenerative potential compared to 2DEVs. By contrast, in the five control groups of depleted CM (3D/2D‐Co/BM‐DEP) and CM, soma (nerve cell body) scarcity was evident, and the neurite outgrowth showed a significant decay with fragmentation effects on the neurites, which can be a sign of nerve damage related to demyelination.^[^ ^43^ ^]^ Neurite elongation was quantitatively analyzed using Neurolucida software, with neurites traced and measured following established protocols.^[^ ^10^ ^]^ For each treatment group, the five longest neurites per triplicate plate were used to represent treatment effects, yielding a total of 15 data points per group across three biological replicates (Figure 4d‐f). Additionally, the inclusion of both CoEVs and BMEVs enabled a comparative assessment of their neuroregenerative effects. TgV1 neurons exhibited GAP‐43‐positive staining following EV treatment as a marker of regenerative sprouting and growth cones, indicating newly formed nerve branches.^[^ ^44^ ^]^ Relative to control cultures receiving only CM, both EV conditions showed visibly stronger GAP‐43 immunofluorescence along neurites, with the most pronounced signal observed in the 3DEV group (Figure 4). pronounced effect on both neurite elongation and regenerative outgrowth of TgV1 neurons than 2DEVs.

Statistical analysis revealed that 3DCoEVs significantly enhanced neurite outgrowth compared to their 2D counterparts, with measured elongations of 12.64 ± 1.5 and 5.3 ± 0.58 mm, respectively (*p* < 0.05, Figure 4d). Similarly, 3DBMEVs significantly promoted neurite elongation, with an average extension of 10.21 ± 1.24 mm, compared to 2DBMEVs, which induced only 3.0 ± 0.32 mm of elongation (p < 0.05, Figure 4e). Comparison between CoEVs and BMEVs showed that 3DCoEVs enhanced neurite elongation more effectively, but the difference was not statistically significant. Conversely, comparing 2D sources revealed that 2DCoEVs significantly enhanced neurite elongation compared to 2DBMEVs (P < 0.005, Figure 4f). Overall, both CoEVs and BMEVs significantly promoted neuronal growth compared to control, with 3DEVs exhibiting superior effects.

### 2.4 EVs Promote Corneal Nerve Regeneration In Vivo

The effects of EVs from two distinct human MSC sources, each derived from five different donors, were evaluated on corneal epithelial healing and nerve regeneration in a mouse model of corneal epithelial debridement. Corneas were injected subconjunctivally either with EVs from 2D‐ or 3D‐cultured BM‐MSCs or Co‐MSCs (2 × 10^9^ particles per 5 µL), and for the control groups, depleted CM from each of the groups and plain CM with no nutrients were used.

**Figure 5:**
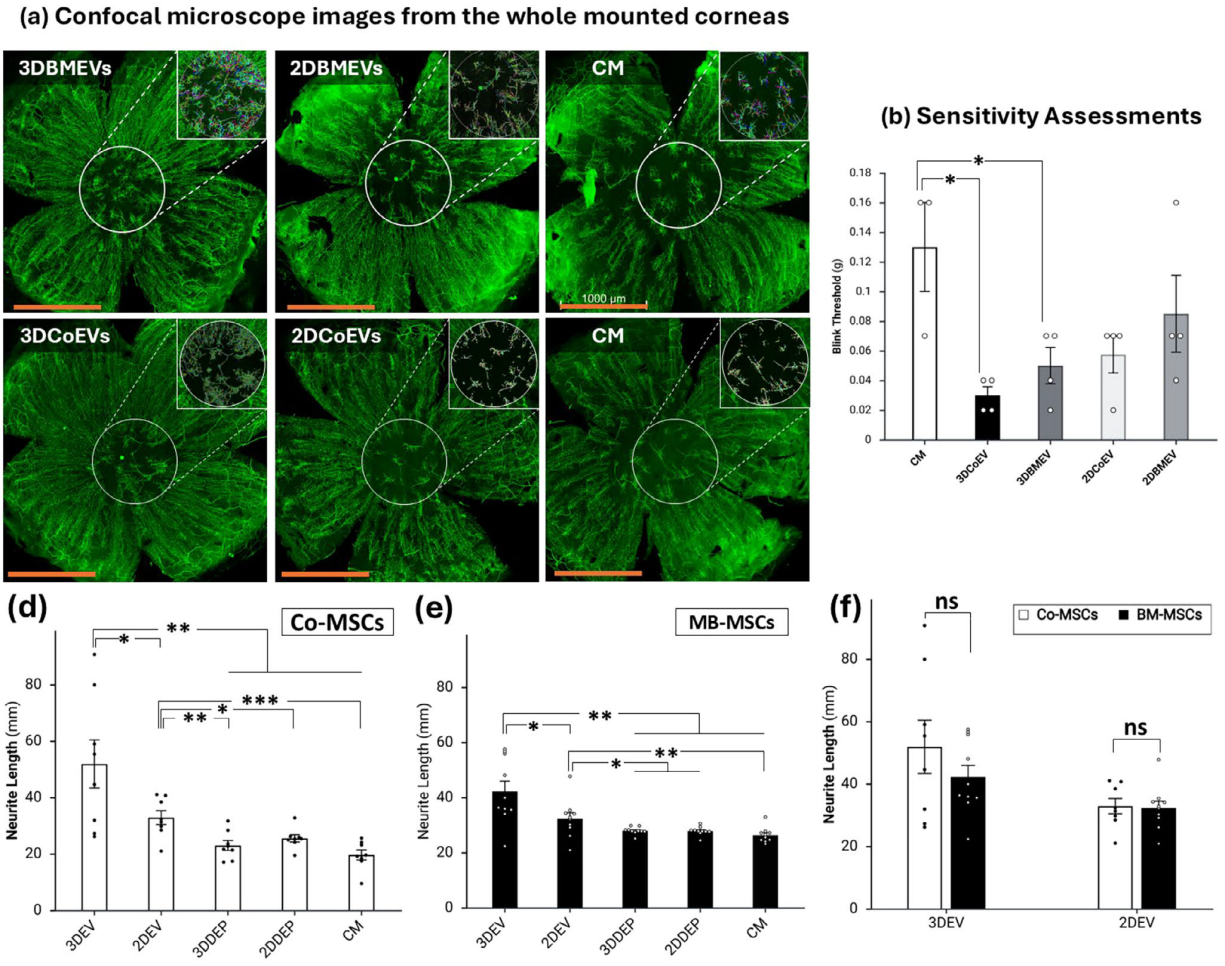
Two weeks following debridement and treatment injections, nerve regeneration was assessed using Neurolucida analysis of corneas stained with βIII Tubulin antibody. a) Nerve fiber density was quantified within a 1 mm central region of the cornea. (a‐inset panels) Inlets depict representative tracings of regenerated nerves, color‐coded and measured using Neurolucida Explorer software. b) Corneas treated with 3DCoEVs demonstrated a statistically significant increase in neuronal growth compared to 2DCoEVs and control groups. c) Corneas treated with 3DBMEVs demonstrated a statistically significant increase in neuronal growth compared to 2DBMEVs and control groups. d) A modest, non‐significant trend toward greater regenerative effects was observed in CoEV‐treated corneas compared to BMEV‐treated corneas. e) Corneal nerve functionality was assessed using von Frey hair filaments. Higher blink thresholds indicated reduced sensitivity. Control corneas exhibited the highest blink thresholds, whereas 3DCoEV‐treated corneas showed the greatest sensitivity. All EV‐treated groups demonstrated improved corneal sensitivity compared to controls. A two‐tailed independent Student's *t*‐test was performed to assess the statistically significant impact of various treatments on nerve regeneration in injured corneas. Scale bar, 1000 µm, n = 4 biological replicates for each condition. CM stands for unconditioned collection media.

The nerve regeneration of the injured cornea was traced and analyzed on the central 1 mm area (**Figure** **5**a). Under physiological conditions, the central cornea harbors a dense and highly organized sub‐basal nerve plexus. However, following 2 mm epithelial debridement injury, the wounded area, including both central and peripheral zones, undergoes substantial denervation. Therefore, restricting analysis to the central 1 mm region provides a focused assessment of regenerative nerve growth, minimizing confounding influences from surviving nerves at the wound periphery. On day 14 post‐injury, control corneas treated with CM alone exhibited a mean nerve length of 23.3 ± 1.2 mm. Treatment with 3D‐derived EVs resulted in significantly greater nerve regeneration compared to controls. Specifically, corneas treated with 3DCoEVs demonstrated a mean nerve length of 51.9 ± 9.1 mm (P = 0.002), while those treated with 3DBMEVs reached 42.2 ± 3.5 mm (P = 0.0006) (Figure 5b,c). Furthermore, when comparing dimensionality, 3DEVs from both tissue sources induced significantly greater nerve growth than their respective 2DEVs, with mean nerve lengths of 32.3 ± 2.1 mm (P = 0.04) and 32.9 ± 2.8 mm (P = 0.03) for 2DCoEV‐ and 2DBMEV‐treated corneas, respectively (Figure 5d). When comparing EVs derived from different tissue sources, 3DCoEVs exhibited a slightly higher regenerative effect compared to 3DBMEVs. However, this difference did not reach statistical significance (Figure 5d).

To evaluate the functionality of regenerated nerves following EV injections, corneal sensitivity was assessed using the blink response test with von Frey filaments.^[^ ^45^ ^]^ Overall, EV‐injected corneas exhibited greater corneal sensitivity compared to controls. Specifically, corneas treated with 3DCoEVs, 3DBMEVs, and 2DCoEVs demonstrated a significantly lower blink threshold than CM‐injected controls, indicating enhanced sensory nerve recovery. Moreover, 3DEVs promoted superior functional outcomes compared to their 2D‐derived counterparts, regardless of tissue source (Figure 5e). A modest trend favoring CoEVs over BMEVs was observed, although differences were not statistically significant. Given the observed functional differences in nerve regeneration between EV groups, we hypothesized that distinct small RNA cargo profiles could mechanistically underlie these divergent outcomes. Therefore, we performed small RNA sequencing to systematically characterize the miRNA composition of Co‐MSC‐ and BM‐MSC‐derived EVs and to explore how culture dimensionality further shapes their molecular profiles.

### 2.5 A Conserved Core miRNA Signature was Detected Across MSC EV Populations

**Figure 6:**
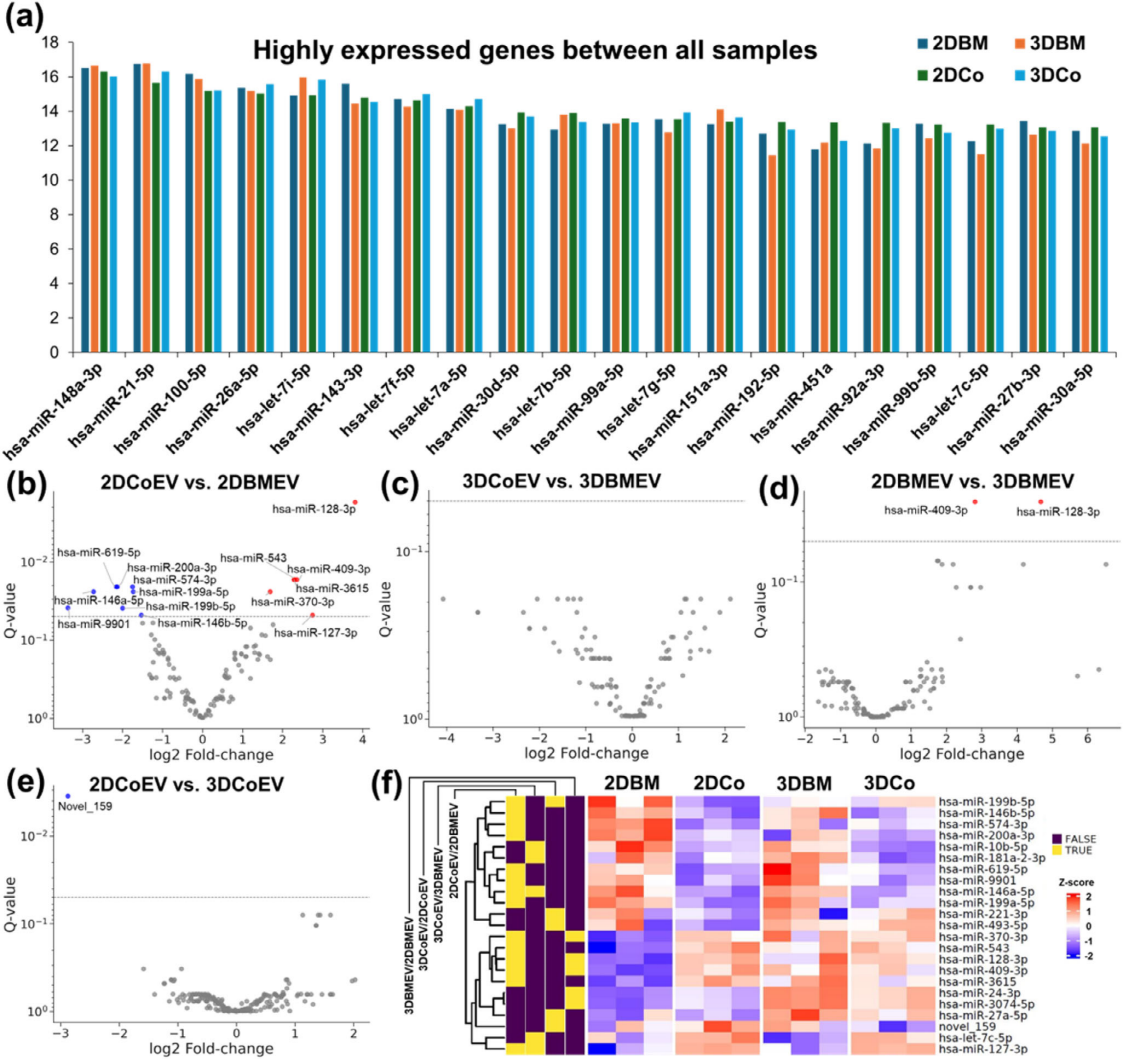
Shared and differentially expressed miRNA signatures among EVs across tissue sources and culture conditions. a) Bar graph showing the top 20 most abundantly expressed miRNAs shared among EVs derived from BM‐ and Co‐MSC cultured under 2D and 3D conditions. b) Comparison between 2DCoEVs and 2DBMEVs reveals the highest number of significantly differentially expressed miRNAs, indicating pronounced molecular divergence under 2D culture conditions. c) Comparison between 3DCoEVs and 3DBMEVs shows a dramatic reduction in differentially expressed miRNAs, suggesting greater molecular similarity between tissue sources in 3D culture. d) Comparison between 3DBMEVs and 2DBMEVs shows significantly upregulated miRNAs, indicating that dimensionality markedly influences the EV cargo from BM‐MSCs. e) In contrast, comparison between 3DCoEVs and 2DCoEVs identifies only one significantly altered miRNA (hsa‐miR‐159), suggesting relative stability in CoEVs cargo across dimensional conditions. f) Heatmap of top enriched miRNAs in EVs shows distinct clustering by culture condition, with reduced tissue‐specific differences in 3D versus 2D.

To better understand the molecular cargo of EVs derived from both MSC sources, we performed small RNA sequencing on isolated EVs. Out of 223 miRNAs detected across all groups, 127 miRNAs (≈57%) were consistently and highly expressed in all four EV populations, indicating the existence of a core conserved miRNA signature shared across tissue sources and culture conditions. In our analysis, the top 20 most abundantly expressed miRNAs across all EVs included miR‐21‐5p, miR‐148a‐3p, miR‐100‐5p, and several members of the let‐7 family. These miRNAs are well‐known for their roles in fundamental regulatory processes, such as cell proliferation, apoptosis, immune modulation, autophagy, and senescence.^[^ ^46^, ^47^, ^48^, ^49^ ^]^ The consistently high expression of these miRNAs across different EV populations underscores their pivotal role in fundamental biological functions and supports a conserved EV‐mediated regulatory mechanism that is maintained irrespective of tissue origin or culture conditions (**Figure** **6**a). Volcano plot analyses revealed that the comparison between 2DCoEVs and 2DBMEVs exhibited the highest number of significantly differentially expressed miRNAs (q‐value < 0.05), indicating pronounced molecular divergence under 2D conditions (Figure 6b). In contrast, the comparison between 3DCoEVs and 3DBMEVs showed a dramatic reduction in differentially expressed miRNAs, with minimal or no statistically significant changes, suggesting a higher degree of molecular similarity in 3D culture conditions (Figure 6c). Furthermore, when comparing 3D versus 2D EVs within the same source, we observed that BMEVs exhibited a more pronounced shift in miRNA expression compared to CoEVs. Specifically, 3DBMEVs versus 2DBMEVs revealed two significantly highly expressed miRNAs (hsa‐miR‐128‐3p and hsa‐miR‐409‐3p) (Figure 6d), whereas 3DCoEVs versus 2DCoEVs showed only one significantly down‐regulated miRNA (hsa‐miR‐159), indicating relative stability in CoEV cargo across dimensional conditions (Figure 6e). To explore overall expression patterns and sample relationships, we generated a heatmap using the top five enriched miRNAs from each of the four EV populations. Hierarchical clustering of Z‐score normalized values revealed distinct separation between 2D and 3DEVs, and pronounced tissue‐specific differences under 2D conditions. Notably, 2DBMEVs and 2DCoEVs formed the most divergent clusters, while 3DBMEVs and 3DCoEVs exhibited higher inter‐group similarity, supporting the idea that 3D culture conditions may reduce source‐specific variability and promote a more convergent EV miRNA profile (Figure 6f).

**Table** **1** summarizes the top three most enriched miRNAs from each group, comparing 3D versus 2D conditions within EVs. Differentially expressed miRNAs were prioritized based on log_2_ fold change, adjusted p‐value (q‐value), and overall expression level, as these parameters are most indicative of biologically relevant shifts. These candidate miRNAs are likely to underlie the functional differences observed in corneal versus bone marrow regenerative effects.

**Table 1:** The top three enriched miRNAs in EVs were compared within the same tissue source.

Comparisons	ID	Fold change	Q Value	log_2_FC
3DBMEVs vs 2DBMEVs	hsa‐miR‐128‐3p	25.53	0.0255	4.67
	hsa‐miR‐409‐3p	7.02	0.025	2.81
	hsa‐miR‐370‐3p	4.56	0.07	2.19
3DCoEVs vs 2DCoEVs	hsa‐miR‐493‐5p	3.04	0.079	1.6
	hsa‐miR‐221‐3p	2.69	0.079	1.42
	hsa‐miR‐27a‐5p	2.62	0.079	1.39

We performed RT‑qPCR on six highly enriched miRNAs using EVs isolated from both 3D and 2D cultures of bone marrow and corneal MSCs. The results of this direct miRNA quantification exhibited the same trend as our small RNA sequencing, with 3D culture conditions consistently upregulating the expression of these key miRNAs relative to their 2D counterparts. Specifically, in BMEVs, 3D culture resulted in a 29.86 ± 9.05‐fold increase in *hsa‐miR‐128‐3p*, a 10.43 ± 2.11‐fold increase in *hsa‐miR‐370‐3p*, and a 3.22 ± 0.25‐fold increase in *hsa‐miR‐409‐3p* compared to 2D culture. Similarly, for CoEVs, 3D culture led to a 2.55 ± 0.43‐fold upregulation of *hsa‐miR‐221‐3p*, a 1.93 ± 0.27‐fold increase in *hsa‐miR‐493‐5p*, and a 1.84 ± 0.35‐fold increase in *hsa‐miR‐27a‐5p*. These validation data confirm that 3D culture significantly enhances the abundance of specific miRNAs within the EV cargo, providing a molecular basis for the superior regenerative efficacy of 3DEVs.^[^ ^49^, ^50^, ^51^ ^]^

**Figure 7:**
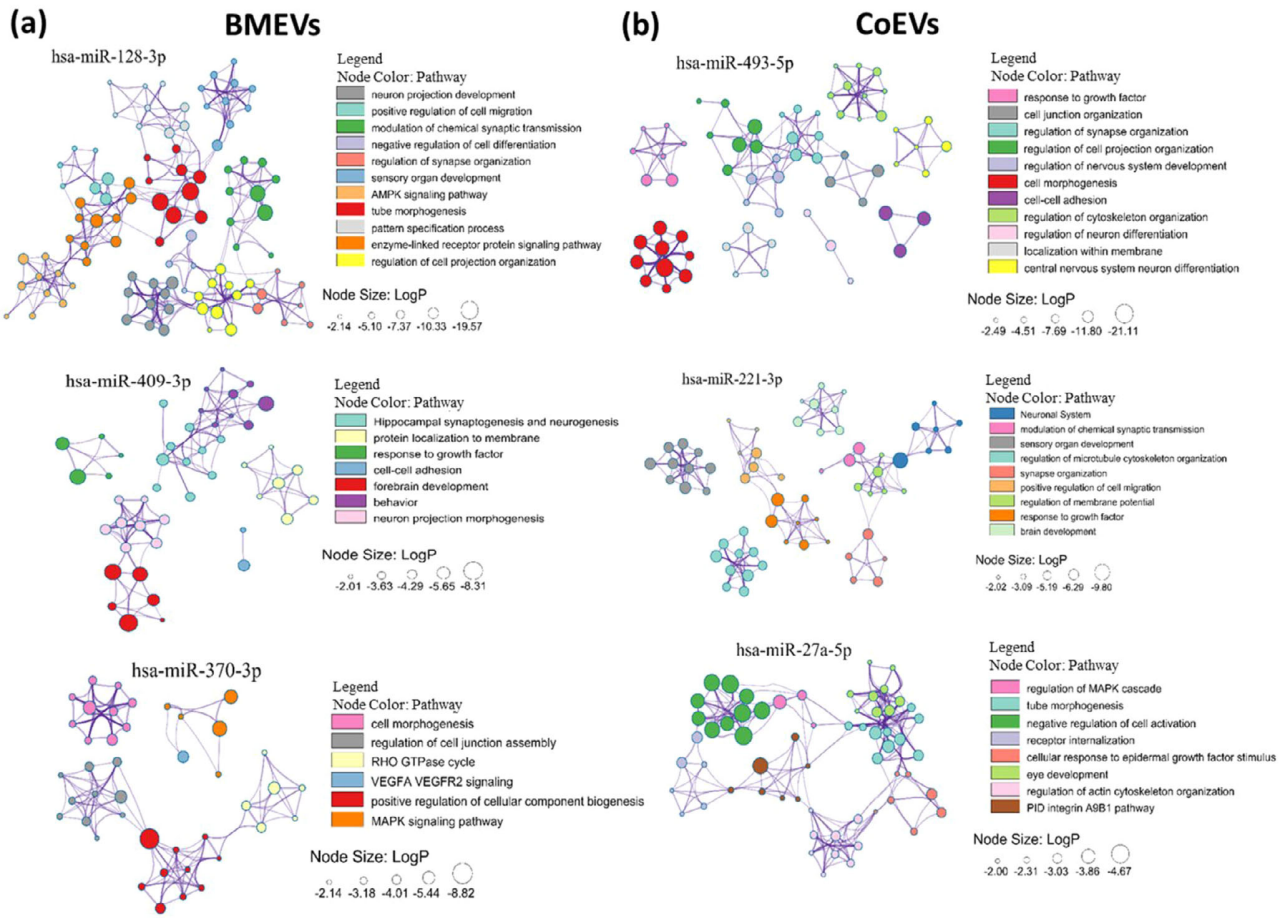
Pathway enrichment networks of target genes for 3D‐enriched miRNAs. a) BMEVs’ miRNAs are associated with neuroplasticity‐related pathways. b) CoEVs’ miRNAs are linked to cytoskeletal regulation and morphogenesis. Node color indicates pathway category; size reflects enrichment significance (LogP).

Predicted target genes of the top‐ranked miRNAs were identified using miRDB, and downstream pathway and gene ontology enrichment analyses were conducted through Metascape to elucidate the biological processes and signaling pathways potentially regulated by these miRNAs. Comparative pathway enrichment analysis of the highly expressed miRNAs miR‐128‐3p, miR‐409‐3p, and miR‐370‐3p in 3D versus 2D BMEVs revealed predominant modulation of signaling pathways associated with synaptic transmission, neuroplasticity, and neuronal survival (**Figure** **7**a).

In contrast, 3DCoEVs, which are enriched in miR‐493‐5p, miR‐221‐3p, and miR‐27a‐5p, exhibit a strong association with structural and developmental processes such as cell adhesion, cytoskeletal reorganization, tissue morphogenesis, and ECM remodeling, axonal guidance (Figure 7b). Together, these findings suggest that BMEVs and CoEVs engage divergent but complementary regenerative mechanisms (functionally versus structurally) that converge to promote comprehensive reinnervation of the cornea.

### 2.6 RT‐qPCR Validation Reveals Distinct Gene Expression Signatures Induced by 3DEV Treatments

To validate pathway‐specific effects of MSC‐derived EVs on TgV1 neurons, we selected two genes associated with neurotrophic signaling and synaptogenesis, including *Ntrk3* and *Syp1*,^[^ ^52^, ^53^ ^]^ and two genes associated with extracellular matrix/cytoskeletal remodeling, *Cdh11* and *Fn1*,^[^ ^54^, ^55^ ^]^ in TgV1 neurons following treatment with 3DBMEVs and 3DCoEVs. BMEV treatment markedly increased neurogenic transcripts, with *Syp1* rising ≈12‐fold (12.75 ± 0.4) and *Ntrk3* approximately sixfold (6.81 ± 0.7) compared with untreated controls. These levels were significantly higher than those observed in CoEV‐treated neurons (*Syp1*: 2.81 ± 0.48, p < 0.0001; *Ntrlk3*: 1.44 ± 0.28, p = 0.004), supporting the preferential activation of synaptogenic and neurotrophic pathways by BMEVs.

**Figure 8:**
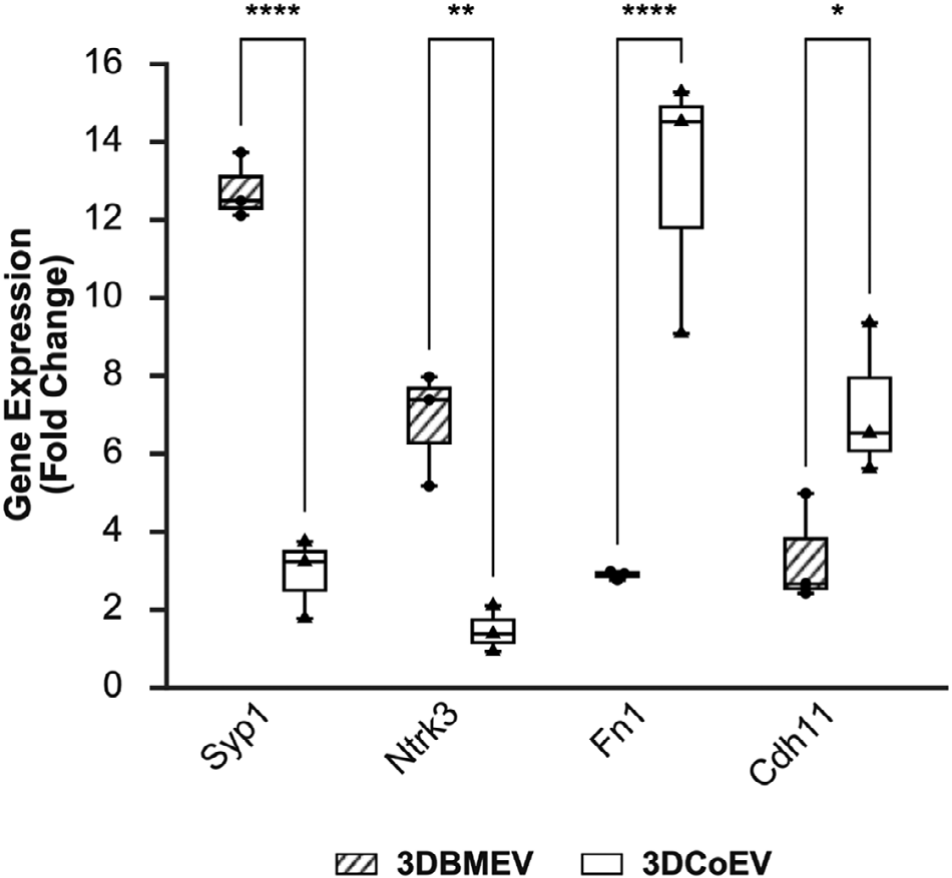
RT‐qPCR validation of neurogenic and matrix‐remodeling gene expression in TgV1 neurons treated with MSC‐EVs from 3D cultures. TgV1 neurons treated with 3DBMEVs exhibited robust upregulation of neurogenic genes, with *Syp1* expression increasing ≈12–14‐fold and *Ntrk3* by 5–8‐fold compared to controls. These levels were significantly higher than those observed in neurons treated with 3DCoEVs, indicating preferential activation of synaptogenic and neurotrophic signaling by BMEVs. Conversely, 3DCoEVs strongly induced the expression of extracellular matrix and adhesion‐related genes, with *Cdh11* and *Fn1* upregulated ≈7‐fold and ≈12‐fold, respectively, significantly exceeding the levels observed in BMEV‐treated neurons. While all EV‐treated groups showed elevated gene expression compared to baseline, the distinct transcriptional profiles highlight the source‐specific mechanistic effects of MSC‐EVs on neuronal remodeling. Data are presented as box‐and‐whisker plots of the mean of biological triplicates with 6 datapoints for each target gene ± SE. Statistical significance was determined by a two‐way ANOVA analysis with Bonferroni multiple comparisons test; ^*^ *p* < 0.05, ^**^ *p* < 0.01, ^****^ *p* < 0.0001.

In addition, CoEVs preferentially upregulated genes associated with extracellular matrix organization and adhesion. *Cdh11* expression was elevated approximately sevenfold (7.14 ± 0.92), and *Fn1* showed a ≈13‐fold increase (12.94 ± 1.59), both significantly surpassing the corresponding expression levels in the BMEV‐treated group (*Cdh11*: 2.62 ± 0.11, *p* = 0.046; *Fn1*: 2.85 ± 0.05, *p* < 0.0001). Although all treatment groups exhibited upregulation relative to baseline, the magnitude and specificity of the gene expression profiles were clearly source‐dependent. (**Figure** **8** and **Table** **2**).

**Table 2:** Relative gene expression between treatment groups and control samples (Mean Fold Change ± SEM) in TgV1 neurons following CoEV and BMEV treatments.

Gene symbol	Treatment group		*p*‐value
	BMEV	CoEV	
*Syp1*	12.75 ± 0.4	2.81 ± 0.48	<0.0001^****^
*Ntrk3*	6.81 ± 0.7	1.44 ± 0.28	0.004^**^
*Cdh11*	2.62 ± 0.11	7.14 ± 0.92	0.046^*^
*Fn1*	2.85 ± 0.05	12.94 ± 1.59	<0.0001^****^

Collectively, these findings demonstrate that BMEVs and CoEVs elicit divergent transcriptional responses in neuronal cells, with BMEVs favoring intrinsic neurotrophic signaling and CoEVs promoting structural remodeling of the extracellular environment. These data provide molecular validation of the functional divergence in EV cargo and support the proposed mechanistic pathways outlined in our study.^[^ ^56^, ^57^ ^]^

## 3 Discussion

MSC‐derived EVs represent a promising cell‐free approach to promote corneal nerve regeneration, an emerging focus in regenerative ophthalmology. In this study, we systematically investigated the neuro‐regenerative capacity of EVs derived from Co‐MSCs and BM‐MSCs, cultured under both 2D and 3D conditions, in accordance with MISEV guidelines.^[^ ^26^, ^58^ ^]^ Polymer‐based precipitation kit for EV isolation was chosen to preserve vesicle integrity and increase efficacy toward isolating exosomes (small vesicles) that are known for their superior therapeutic potential.^[^ ^59^ ^]^ Through in vitro and in vivo assessments, we demonstrate that both tissue origin and culture dimensionality significantly influence EV yield, molecular cargo, and regenerative function. Small RNA sequencing revealed distinct miRNA profiles across conditions, providing mechanistic insight into how EV composition is shaped by source and microenvironment.

Our data show that 3D cultures enhanced EV yield and induced a more diverse tetraspanin profile, including higher expression of CD9 and CD81, which may improve EV bioavailability and uptake. Biophysical characterization using TEM analysis provided morphological validation. Homogeneous population of nanoparticles exhibiting the distinct cup‐shaped and spherical morphology attributed to exosomes.^[^ ^60^ ^]^ Notably, 3DEVs consistently outperformed their 2D counterparts in promoting neurite elongation and corneal nerve regeneration, regardless of MSC source. This effect likely stems from the biomimetic features of 3D culture, which recreate critical mechanical and biochemical cues that influence EV biogenesis and cargo loading.^[^ ^12^, ^44^, ^61^ ^]^ Although CoEVs exhibited a modest but consistent functional advantage over BMEVs in both 2D and 3D settings, the differences were most evident in 2D culture, suggesting that dimensionality may attenuate source‐specific variability by harmonizing MSC paracrine signaling.

The divergent developmental origins of MSC populations likely contribute to the distinct functional effects observed in their EVs,^[^ ^62^, ^63^, ^64^ ^]^ Co‑MSCs arise from the neural crest, a transient embryonic population that gives rise to the trigeminal ganglion, craniofacial mesenchyme, and corneal stroma.^[^ ^62^, ^63^, ^64^, ^65^, ^66^ ^]^ Neural crest cells are characterized by their remarkable motility and dynamic regulation of cell–cell and cell–matrix adhesion molecules, such as cadherins and fibronectin, during epithelial‑to‑mesenchymal transition and tissue morphogenesis.^[^ ^67^ ^]^ This lineage retains an epigenetic memory that favors extracellular matrix (ECM) remodeling and adhesion regulation, consistent with the robust upregulation of *Cdh11* and *Fn1* observed in CoEV‑treated neurons. In the corneal niche, this predisposition may allow Co‑MSC‑derived EVs to indirectly promote regeneration by establishing a permissive extracellular environment that supports epithelial integrity and nerve reinnervation.

In contrast, bone marrow MSCs (BM‑MSCs) originate from the mesodermal lineage, where they serve as stromal regulators of hematopoiesis and exhibit broad transcriptional and phenotypic plasticity in response to environmental cues.^[^ ^12^, ^68^ ^]^ Under 3D culture conditions, BM‑MSCs undergo substantial reprogramming that enhances their neuroregenerative potential, as demonstrated by the enrichment of miR‑128‑3p and miR‑409‑3p, microRNAs implicated in axonal guidance, synaptic signaling, and neurotrophic activity.^[^ ^69^, ^70^ ^]^ Consistent with these findings, BMEVs in our study significantly upregulated *Syp1* and *Ntrk3* in TG neurons, reflecting a more direct activation of neurotrophic pathways. Together, these lineage‑driven distinctions suggest that CoEVs facilitate corneal regeneration primarily through extracellular and structural modulation, whereas BMEVs exert intrinsic neurotrophic and synaptogenic effects, mediated by plasticity‑associated miRNA cargo and signaling pathways.

Furthermore, both in vitro neurite outgrowth and in vivo sub‐basal nerve regeneration correlated with miRNA cargo profiles. While 2DCoEVs and 2DBMEVs showed the largest divergence in miRNA signatures, 3DCoEVs and 3DBMEVs exhibited notable molecular convergence. These findings suggest that a 3D culture not only improves EV output but also reduces source‐dependent variability, enhancing scalability and reproducibility in therapeutic applications. Heatmap clustering and pathway enrichment analyses further support that CoEVs predominantly promote structural and immunomodulatory processes (e.g., ECM remodeling, cell junction assembly), while BMEVs activate neurotrophic pathways (e.g., PI3K/Akt, MAPK).

Beyond structural regeneration, functional recovery was supported by corneal sensitivity testing, with 3DEVs producing the most pronounced effects. Given that sensory restoration remains a major unmet challenge in neurotrophic keratopathy and other neuropathies,^[^ ^43^ ^]^ our results underscore the translational relevance of EV therapy. The immunomodulatory and anti‐inflammatory cargo of EVs may contribute to this effect, promoting a regenerative microenvironment conducive to nerve survival and integration.^[^ ^71^, ^72^, ^73^ ^]^

Our small RNA‐seq data revealed a significant upregulation of miR‐128‐3p in BMEVs and miR‐221‐3p in CoEVs derived from 3D cultures compared to 2D conditions, underscoring the influence of dimensionality on EV cargo composition. These findings are consistent with previous studies showing that miR‐128 plays a pivotal role in axonal regeneration and synaptic remodeling,^[^ ^74^ ^]^ while miR‐221 is known to promote neurite outgrowth and neuronal survival.^[^ ^75^ ^]^ The enrichment of these miRNAs in our 3DEVs offers a compelling molecular explanation for the enhanced neuro‐regenerative effects observed in both in vitro and in vivo models. Importantly, these effects were more pronounced in BMEVs, which exhibited substantial shifts in miRNA profiles between 2D and 3D cultures. In contrast, CoEVs displayed relatively stable miRNA content across culture conditions. This stability may reflect a more fixed epigenetic program or intrinsic adaptation to the ocular niche, whereas the greater transcriptomic plasticity observed in BMEVs suggests heightened responsiveness to environmental cues. These molecular differences likely mirror the biological roles and demands of their native tissue environments. In particular, the corneal niche is highly specialized and exposed, requiring precise epithelial‐neuronal coordination to maintain barrier integrity, immune privilege, and sensory function. These findings support a model in which niche regulation plays a decisive role in enabling and sustaining nerve regeneration for corneal neurons, whose terminal projections are directly exposed to the external environment.^[^ ^76^, ^77^, ^78^ ^]^ The ability of CoEVs to modulate the epithelial‐neuronal interface, maintain barrier function, and fine‐tune local immune responses may provide critical permissive signals that precede or amplify the effects of neurotrophic support. Thus, while BMEVs primarily activate intrinsic neuronal growth machinery, the contextual precision of CoEVs in remodeling the ocular surface niche may be a key determinant of effective corneal nerve repair. This suggests that microenvironmental priming may be a rate‐limiting step in regeneration, and future therapeutic strategies may benefit from leveraging niche‐adapted EVs or combinatorial approaches that engage both intrinsic and extrinsic pathways. Together, these observations suggest that while both tissue source and dimensionality influence regenerative efficacy, the enhanced neural outcomes associated with 3D culture are mechanistically supported by the selective enrichment of functionally relevant miRNAs. This dual‐regeneration strategy, CoEVs supporting structural remodeling and niche adaptation, and BMEVs enhancing neuronal intrinsic growth, suggests potential synergy in combinatorial approaches. Future studies may explore co‐delivery strategies or sequential EV administration to capitalize on these complementary mechanisms.

Despite their divergent primary mechanisms of action, both BMEVs and CoEVs led to a significant increase in the expression of *Ntrk3*, *Syp1*, as well as *Cdh11* and *Fn1*. These upregulations are indicators that axonal elongation is being followed by the successful establishment of presynaptic terminals and remodeling of the extrinsic niche to overcome physical and molecular barriers to regeneration.^[^ ^56^, ^79^ ^]^ This finding serves as a crucial point of convergence for the two distinct pathways. It demonstrates that whether regeneration is driven by enhancing the neuron's intrinsic responsiveness to growth factors (the BMEV model) or by engineering a more permissive extrinsic environment (the CoEV model), the ultimate and functionally relevant outcome is the restoration of neural circuitry. This shared endpoint underscores that multiple effective strategies exist for promoting nerve repair, and the specific molecular route taken is dictated by the unique bioactive cargo of the therapeutic EVs.

### 3.1 Statement of Limitations

While our study provides a strong foundation for EV‐based nerve regeneration, several important questions remain for future investigation. Our primary aim was to evaluate how tissue source and culture dimensionality influence EV efficacy. Although our findings revealed consistent trends across five independent donors, mechanistic confirmation of miRNA function remains essential. We are currently conducting proteomic analyses, which have revealed cargo differences mirroring the small RNA data. Future studies will integrate multi‐omics data and employ targeted gain and loss of function experiments to validate the contributions of specific miRNAs and proteins to regenerative outcomes. In terms of delivery, we opted for subconjunctival injection based on our prior dose optimization studies, which demonstrated robust effects with a single dose. While this method is more invasive than topical administration, it enables targeted delivery to the corneal stroma, reducing losses from tear drainage and blinking. Nevertheless, delivery route comparisons will be explored in future work to balance efficacy and clinical practicality. Moreover, although our study focused on a 14‐day endpoint, extended follow‐up (30‐60 days) will be necessary to assess the durability of nerve repair. Despite donor‐to‐donor variability, a known challenge in MSC‐based therapeutics, the superior performance of 3D EVs remained consistent, supporting the reproducibility and scalability of our findings.

In summary, this study establishes a strong foundation for EV‐based corneal nerve therapies and highlights how dimensionality and tissue origin shape the regenerative potential of EVs and offers a blueprint for developing niche‐adapted, bioactive EV therapies for corneal and potentially broader neural repair applications. Our findings provide critical insights into the design of next‐generation EV products with enhanced therapeutic potency and translational relevance.

## 4 Conclusion

This study demonstrates that MSC‐derived EV therapy holds great promise for corneal nerve regeneration, with both tissue origin and culture dimensionality critically shaping therapeutic efficacy. CoEVs, shaped by their neural crest lineage and ocular surface niche, exhibited a stable, niche‐adapted cargo profile enriched in miRNAs involved in structural remodeling and immunomodulation, thereby promoting a permissive regenerative microenvironment. In contrast, BMEVs displayed greater transcriptomic plasticity under 3D conditions, enriched with miRNAs that activate classical neurotrophic signaling cascades, and suggest a more neuron‐intrinsic mechanism of action. Notably, 3D culture enhanced EV yield and normalized cargo variability, offering a scalable strategy for consistent therapeutic production. Functional assessments revealed that while both EV types supported neurite outgrowth and sensory recovery, CoEVs consistently yielded superior outcomes, particularly in restoring corneal sensitivity, a clinically relevant endpoint. These findings underscore the importance of aligning EV source and culture conditions with the biological demands of the target tissue. Moving forward, regenerative strategies that couple niche‐specific EVs with optimized 3D culture systems may hold substantial promise for advancing EV‐based therapies in neuro‐ophthalmology and broader neurological applications.

## 5 Experimental Section

### Cell culture

Corneal and bone marrow stem cells were each obtained from 5 different donors. The primary Co‐MSCs were obtained from Dr. Ali R. Djalilian's (Chicago, IL) laboratory. Primary Co‐MSCs were isolated from the corneoscleral rim and have been previously characterized and validated as functional MSCs.^[^ ^80^, ^81^, ^82^ ^]^ The BM‐MSCs were obtained from RoosterVial‐hBM‐XF (RoosterBio Inc., MD) and Dr. Peiman Hematti's laboratory (Milwaukee, WI), isolated from the filters remaining after bone marrow harvest from healthy donors, and have been characterized and validated previously as functional MSCs.^[^ ^83^, ^84^ ^]^ All primary cells were obtained in passage one and were seeded into T75 flasks and grown to 80–90% confluence in growth media containing RoosterBasal MSC‐CC media supplemented with 2% RoosterBooster‐MSC‐XF (RoosterBio Inc., MD) and 1% antibiotic‐antimycotic (Corning, Glendale, AZ). This growth media is approved for cGMP EV manufacturing across diverse cell and tissue types and follows the guidelines of minimal information for studies of extracellular vesicles (MISEV).^[^ ^26^ ^]^ Cells were then gently washed three times with phosphate‐buffered saline (PBS) (Corning, Glendale, AZ) and incubated at 37 °C in 5% CO_2_. All cells in this study were used in passages #3‐5.

### 5.1 Cultures for EV Production

T225 flasks were seeded with 13 000 cells cm^−2^ and incubated until they achieved a confluency of ≈80%. The growth media was decanted from the flasks, and cells were gently washed with PBS three times. Then, 45 mL serum‐free, phenol red‐free RoosterCollect‐EV media was added to flasks for 72 h to induce EV release. Finally, the secretome containing the exosomal particles was collected from the flasks and used to isolate 2D‐obtained EVs (2DEVs). The particles obtained from 2D‐cultured Co‐MSCs and BM‐MSCs were named 2DCoEV and 2DBMEV, respectively.

### 5.2 Cultures for EVs Production

The cells were cultured in a Vertical‐Wheel Bioreactor (PBS Biotech, Inc., CA) following a previous study.^[^ ^12^ ^]^ The cells were detached from T75 flasks, and 2 million cells were counted and seeded into a vertical wheel bioreactor vessel containing 1.25 g of preincubated Low Concentration Synthemax II Microcarriers (Corning, Glendale, AZ) in growth media. The culture was incubated for 20 min without agitation, and the flask was then swirled gently to redistribute the non‐adherent cells. The flask was then incubated for another 10 min without agitation. Next, 70 mL of growth media was added to obtain a 3D culture system with 1.25 g of microcarriers, 2 million cells, and 90 mL of growth media. The wheel of the bioreactor was set to rotate at a speed of 25 rpm, and the bioreactor was left in the incubator for 73 h. On the third day, a 3 mL sample was taken from the culture to count the cells, and 1.5 mL RoosterReplenish‐MSC‐XF (RoosterBio Inc., MD) dissolved in 1.5 mL growth media was added to the culture. The wheel rotation speed was set to 35 rpm until day 5, when the growth media was removed from the culture cautiously, and the microcarriers (bearing the cells) were washed with PBS three times. The cells were incubated in 45 mL of unconditioned collection media (CM) for another 72 h to induce EV release from the cells. Finally, the conditioned media/secretome containing the exosomal particles was collected from the Bioreactor carefully and passed through a 100 µm cell strainer to remove the microcarriers. The secretome was then used to isolate 3D‐obtained EVs (3DEVs). The particles obtained from 3D‐cultured Co‐MSCs and BM‐MSCs were named 3DCoEV and 3DBMEV, respectively.

### Isolation of MSC‐Derived EVs

After obtaining the secretome from both culture systems, it was sequentially centrifuged at 500 ×g for 15 min at 4 °C to remove cells, followed by 2000 ×g for 10 min at 4 °C to eliminate cell debris and larger particles. The clarified secretome was then subjected to EV isolation using two methods: **I) Ultracentrifugation (UC)**: The supernatant was further centrifuged at 10 000 ×g for 30 min at 4 °C to remove large vesicles, followed by 100000 ×g for 90 min at 4 °C to pellet EVs. The pellet was washed in phosphate‐buffered saline (PBS), UC again at 100 000 ×g for 90 min, and resuspended in 200 µL double‐filtered (0.22 µm) PBS before storage at ‐80 °C for downstream applications. **II) Precipitation‐Based Isolation**: The purified secretome was mixed with Total Exosome Isolation Reagent (Thermo Fisher, MA) at a 2:1 ratio (secretome to reagent) and incubated at 4 °C overnight. The mixture was centrifuged at 10 000 ×g for 1 h at 4 °C to precipitate EVs, which were then resuspended in 200 µL double‐filtered (0.22 µm) PBS and stored at −80 °C. The reagent facilitates EV precipitation by reducing the aqueous solvating function, allowing less‐soluble components such as EVs to be efficiently pelleted via low‐speed centrifugation. The supernatant was aspirated and stored at −80 °C as the EV‐depleted secretome to be used as control groups (2DCoDEP, 2DBMDEP, 3DCoDEP, and 3DBMDEP). For all subsequent functional, molecular, and characterization experiments, EVs were isolated using the precipitation‐based commercial kit to ensure the preservation of vesicle integrity and function.

### EV Size Analysis and Quantification

EV size and concentration were assessed utilizing the NanoSight NS300 (Malvern Panalytical, UK) using an enhanced particle size distribution algorithm with a high‐resolution finite track length adjustment (FTLA). Briefly, all samples were pre‐diluted in double‐filtered (0.22 µm filters) PBS.^[^ ^85^ ^]^ The camera level was adjusted to achieve a distinct visualization of all particles, following the manufacturer's recommended settings (NanoSight NS300 User Manual, MAN0541‐01‐EN‐00, 2017). For each measurement, samples were passed over a laser with a pump speed of 100 µL/s for 30 s and repeated three times with a 2‐s interval, and videos were captured at 25 °C. After capture, the videos were analyzed by the in‐built NanoSight Software NTA 3.1 Build 3.1.46 with a detection threshold of 5. The motion of the particles is tracked and related to size using the Stokes‐Einstein equation.

### EV Characterization– Single Particle Interferometric Reflectance Imaging Sensor (SP‐IRIS) (ExoView)

SP‐IRIS experiments were conducted at the core facility of the University of Pennsylvania, identified by the Research Resource Identifier (RRID): SCR_022444. Tetraspanins CD9, CD63, and CD81 distribution was analyzed using the ExoViewR100 platform, utilizing the Leprechaun human tetraspanin ExoView kits (Unchained Labs Catalog number 251–1044) following the kit assay protocol.^[^ ^12^ ^]^ Briefly, EV prep was diluted 1000 times with the incubation solution II. The dilute samples (50 µL) were placed on top of the chips inside a Falcon 24‐well cell culture plate, flat bottom (Fisher Scientific Catalogue number 08‐772‐1) for the capture of EV carrying mouse anti‐human CD9 (Clone HI9a), mouse anti‐human CD81antigen (Clone JS‐81), mouse anti‐human CD63 (clone H5C6) and mouse isotype IgG1, and control (Clone MOPC‐21). Wells were sealed with adhesive plate seals to prevent evaporation. After 16 h incubation at room temperature (RT), chips were washed 3 times with 1X solution A for 3 min on an ELISA microplate orbital shaker at 500 rpm (Fisherbrand Fisher Scientific Catalogue number 88‐861‐023). Chips were then incubated with an antibody cocktail made of 0.6 µL mouse anti‐human CD81 (Clone JS‐81) conjugated with Alexa Fluor 555, 0.6 µL mouse anti‐human CD63 (Clone H5C6) conjugated with Alexa Fluor 647, and 0.6 µL of mouse anti‐human CD9 (Clone Hi9a) conjugated with Alexa Fluor 488 in 300 µL of blocking solution for 1 h at RT on an orbital shaker at 500 rpm. Chips were washed 1 time with 1X solution A, 3 times with 1X solution B, and 1 time with DI water, respectively, for 3 min at 500 rpm. After careful drying of the chips, image acquisition from each chip was carried out using the ExoView R100 platform, and the data were analyzed by the ExoView Analyzer software version 3.2 (NanoView Biosciences). The images of the acquisition were visually inspected, and all the artifacts in the spots were manually removed from the analysis. Non‐specific binding was checked on the mouse isotype control IgG spots. The cut‐off was manually established for all the chips to exclude the majority of the signal (>95%) captured on the isotype control. In accordance with best practices for antibody‐based EV profiling, including ExoCheck/ExoView platforms, isotype controls were matched to the capture antibody subclasses (e.g., IgG1, IgG2a) but lacked specificity for EV markers. Signals detected on the IM control spots were consistently low across all samples and were used to determine the background binding threshold. Only signals exceeding the IM‐derived background were considered specific and included in the quantification of EV subpopulations. This approach ensured unbiased artifact exclusion and consistency across all experimental chips.

### Western Blotting

Immediately after isolation, high concentrations of EV solutions (≈10^8^ particles, 20 µL) were suspended in a lysis buffer (150 µL, CelLytic M, Sigma Aldrich, St. Louis, USA) with protease and phosphate inhibitors (1.5 µL, Thermo Fisher) mixed with Ethylenediaminetetraacetic acid (EDTA, 1:1 ratio). A T75 flask was used to culture Co‐MSCs up to 80% confluence and obtain protein lysates. 1 mL of lysis buffer plus protease and phosphate inhibitor with EDTA was added to the flask, and cells were scraped off. The cell suspension was centrifuged at 13000 ×g for 15 min at 4 °C to separate the supernatant (lysates) from the cell debris. NanoDrop was used to measure the protein content of each lysate batch and to ensure the same amount of protein was used for each Western blot (WB) analysis. Reducing agent and LDS sample buffer were added to each sample lysate accordingly, and the solutions were heated up to 70 °C for 10 min. Samples were loaded into a 4–12% SDS‐polyacrylamide gel and subjected to gel electrophoresis (PAGE) at 100 volts for 1 h. Gels were then transferred to ethanol‐activated PVDF membranes using a wet transfer protocol. Membranes were then blocked with non‐fat milk in TBS‐T for 1 h before incubating with ALIX, GAPDH, and Calnexin antibodies resuspended in 2% BSA/TBS‐T overnight (anti‐calnexin and anti‐GAPDH both sourced from Abcam BioTech. Co., Cambridge, UK, 1:1000 dilution). Membranes were washed in TBS‐T, incubated with HRP‐conjugated secondary antibodies (Cell Signaling Technologies, 1:1000), and resuspended in 2% BSA/TBS‐T. They were then washed in TBS‐T, visualized using SuperSignal West Femto Maximum Sensitivity Substrate (Thermo Fisher), and scanned using iBright ECL1500 (Thermo Fisher).

### Transmission Electron Microscopy (TEM)

Isolated EVs were analyzed morphologically using TEM using previously established methods with minor modifications.^[^ ^86^ ^]^ A 5 µL aliquot of each EV preparation was first fixed in a 2% paraformaldehyde solution. The fixed samples were then adsorbed onto Formvar carbon‐coated electron microscopy grids and incubated for 30 min at room temperature in a desiccated environment. Subsequently, the grids were post‐fixed with 1% glutaraldehyde for 5 min, followed by a series of eight washes in distilled water, each lasting 2 min. For contrast enhancement, the samples were treated with 2% uranyl‐oxalate for 5 min. Finally, the grids were embedded in a chilled mixture of 4% uranyl acetate and 2% methylcellulose for 10 min on ice. Imaging was performed using a JEOL JEM 1011 transmission electron microscope (JEOL Ltd., Tokyo, Japan) to visualize the EVs.

### Animals

All experiments were performed according to the guidelines of the Association for Research in Vision and Ophthalmology Statement for the Use of Animals in Ophthalmic and Vision Research, in compliance with the Animal Research: Reporting of In Vivo Experiments (ARRIVE) guidelines. All animal experiments were approved by the Institutional Animal Care and Use Committee from the University of Illinois‐Chicago under the approved protocol 23–107, following the US NIH Guide for Care and Use of Laboratory Animals.

### TgV1 Neuronal Growth Assay

Briefly, 4–6‐week‐old wild‐type C57BL/6J mice were euthanized, and the V1 branch of the trigeminal ganglia (TgV1) was isolated from the cranial cavity. Juvenile mice (4–6 weeks old) were specifically chosen for these in vitro cultures, as tissue from this age yields a higher number of viable neurons with more robust neurite outgrowth, thereby improving the reproducibility of the assay. The tissue was then subjected to sequential enzymatic digestion with papain solution (Worthington), followed by type II collagenase (Worthington, Lakewood, NJ) and type II dispase (Thermo Fisher) mix to dissociate ganglia into a single‐cell suspension. The resulting neurons were subsequently cultured on 35 mm tissue culture dishes containing a 20 mm glass‐bottom well (Cellvis, Mountain View, CA) coated with poly‐D‐lysine. TgV1 cells were cultured in Neurobasal‐A medium supplemented with B27 and antibiotics for two days to observe minimal neurite growth. After the onset of neurite growth, the growth media was gently removed, and the dishes were washed with PBS twice before being supplemented with CM. Each dish was treated with one of the four treatment groups (2D/3D‐Co/BM‐EVs) with an equal number of particles (2 × 10⁹ EVs/dish), or received one of the four control groups consisting of depleted conditioned media (2D/3D‐Co/BM‐DEP), in which extracellular vesicles were removed by ultracentrifugation while preserving soluble, non‐vesicular components. Untreated dishes served as an additional negative control. Neurons were monitored for 2 days for cell viability and neurite growth, then fixed in 4% paraformaldehyde (PFA) and stained with β III tubulin at 1:400 dilution (Abcam BioTech. Co., Cambridge, UK). GAP‐43 antibody staining at 1:2000 dilution (Bio‐Techne, Minneapolis, MN) was also utilized as a marker of regenerative sprouting and growth cones, indicating newly formed nerve branches. The experimental design was repeated five times, separately, for each cell type.

### TgV1 Neurite Length Analysis

Treatment conditions were masked, and the total number of neurons was counted on each dish. Neurons were grouped into four distinct categories: no neurites, small neurites (<25% image occupied at 20× magnification), medium neurites (25–75%), and large neurites (75–100%). Five regions of interest, each 1×1 mm, were selected for each treatment, and neurons within these regions were imaged at 10× magnification using a fluorescent microscope (EVOS M5000, ThermoFisher). Neuronal growth was traced using Neurolucida, and the total length of neurites was extrapolated onto Excel.

### Corneal Epithelial Debridement Model

An in vivo corneal defect model in mice was implemented in this study following previously reported protocols with slight modifications.^[^ ^10^, ^87^ ^]^ For EVs from each cell type, a group of 10–12 week‐old wild‐type C57BL/6J mice was anesthetized by a mixture of 0.9 mL Ketamine and 0.5 mL Xylazine in 8.6 mL PBS, followed by administration of a local proparacaine drop (0.5%) on the injured eye. Adult mice (10–12 weeks old) were used for the in vivo model to ensure a mature ocular surface and immune environment, avoiding confounding variables from ongoing postnatal corneal nerve maturation. Once complete anesthesia was reached, animals were subjected to a 2 mm central corneal epithelial debridement with removal of the sub‐basal nerve plexus without damaging the underlying stroma layer using a 2 mm biopsy punch for marking the wound area and an AlgerBrush II with a 0.5 mm Burr (Precision Vision, IL) to remove the epithelium. Each animal received a 5 µL subconjunctival injection dose (containing 2 × 10^9^ EV particles) in its right eye. The injections were randomly distributed among the groups with an equal number of male and female animals. The experiment was replicated 3 times for each of the two groups of treatments obtained from BM‐MSCs or Co‐MSCs. The 9 injection groups in each experiment set were 2D‐ or 3D‐derived EVs, 2D‐ or 3D‐derived depleted media, and CM. Corneal wound healing was monitored using slit‐lamp microscopy with fluorescein staining at 18–20 and 42–44 h post‐debridement. After 14 days, all animals were humanely euthanized, and after the enucleation of their eyes, their corneas were dissected, stained, and whole mounted for nerve regeneration analysis. We conducted three independent experiments, and in each experiment, we used n = 27 mice, 10–12 weeks old. An equal number of males and females were used for three repeats of the 9 injection groups, including 4 EV treatments, 4 depleted CM control groups, and 1 plain CM control injection. The total number of animals used in this study was n = 81.

### Corneal Whole Mount and Immunofluorescence Staining

Corneas were dissected from harvested eyes in a PBS bath and immediately fixed in 100% acetone on ice for 30 min. The corneas were then blocked at RT for 1 h using a blocking buffer of 1% BSA, 0.25% Triton X‐100, and 2.5% donkey serum. Afterward, tissues were stained with rabbit anti‐β III tubulin antibody overnight at 4 °C, followed by Alexa Fluor 488‐conjugated goat anti‐rabbit IgG (Abcam BioTech. Co., Cambridge, UK) at RT for 1 h. Corneas were then flat‐mounted using a mounting medium (Vector Laboratories, Inc., CA) and imaged at 10× under a fluorescent microscope (EVOSTM 5000, ThermoFisher) to obtain focused images on the sub‐basal plexus while excluding stromal nerve bundles from the Z‐stack images. Nerve regeneration analysis was conducted using Neurolucida software to study the neurite length in a central corneal circle using a 1 mm diameter contour.

### Corneal Nerve Sensitivity Assessment

The calibrated von Frey Hairs (North Coast Medical) method was used to evaluate the sensitivity and functionality of the affected corneas after injections (4 EV treatment groups and 1 CM injected group). Based on previous studies on unanesthetized mice, we held the animals under a microscope and immediately tapped the center of the cornea with thin filaments with ascending stiffness (0.008–0.4 g).^[^ ^88^ ^]^ Blink thresholds were recorded as minimal force (g) to elicit at least two blink responses from three touches with 10 s intervals. Three mice were assessed in each injection group.

### TgV1 Neuron Culture and EV Treatment for RNA Analysis

TgV1 neurons were isolated from 4–6‐week‐old wild‐type C57BL/6J mice and cultured as described previously in the TgV1 neuronal growth assay section. Following an initial 48 h culture period for neurite initiation, cells were assigned to one of three groups: untreated control, 3DCoEV, or 3DBMEV treatments. EV treatments were administered at a final concentration of 2 × 10^9^ EVs/dish, consistent with the concentrations used in functional assays. After a 48 h incubation period with the respective treatments, cells were harvested for total RNA isolation.

### Total RNA Isolation and Quality Control

Total RNA was extracted from the cultured TgV1 neurons using the Invitrogen mirVana miRNA Isolation Kit, with phenol (Thermo Fisher Scientific, Waltham, MA) according to the manufacturer's protocol. Following extraction, the concentration and purity of the isolated RNA were determined using a NanoDrop 2000 spectrophotometer (Thermo Fisher Scientific, Waltham, MA). RNA quality was assessed by ensuring the A260/A280 ratio was between 1.9 and 2.1.

### cDNA Synthesis and Quantitative Reverse Transcription quantitative Polymerase Chain Reaction (RT‐qPCR)

Complementary DNA (cDNA) was synthesized from 100 ng of total RNA per sample using the High‐Capacity cDNA Reverse Transcription Kit (Applied Biosystems, Foster City, CA) following the manufacturer's instructions.

Quantitative real‐time PCR was performed using the PowerUp SYBR Green Master Mix (Applied Biosystems) on a QuantStudio 5 Real‐Time PCR System (Applied Biosystems). Each 10 µL reaction consisted of 5 µL of 2X Master Mix, 2.5 µL of forward primer (1 µM), 2.5 µL of reverse primer (1 µM), and 0.1 µL of diluted cDNA template. The expression of the following murine genes was quantified: Neurotrophic Receptor Tyrosine Kinase 3 (*Ntrk3*), Synaptophysin 1 (*Syp1*), Cadherin 11 (*Cdh11*), and Fibronectin 1 (*Fn1*). PCR primer sequences were retrieved from the PrimerBank website.^[^ ^89^ ^]^ All the DNA oligonucleotide primers were synthesized by Sigma Aldrich. Beta‐actin (*Actb*) was used as the endogenous control for normalization. The thermal cycling protocol consisted of an initial denaturation step at 95 °C for 10 min, followed by 40 cycles of denaturation at 95 °C for 10 s and annealing/extension at 60 °C for 30 s. A melt curve analysis was performed at the end of each run to verify the specificity of the amplified product. All reactions were performed in triplicate for each biological sample.

### RT‐qPCR Data Analysis

Relative gene expression was quantified using the comparative Ct (2^−ΔΔCt^) method. The threshold cycle (Ct) value for each target gene was normalized to the Ct value of the endogenous control, *Actb*, to obtain the ΔCt value (ΔCt = Ct_target_−Ct_Actb_). The ΔΔCt was then calculated by subtracting the average ΔCt of the untreated control group from the ΔCt of each treated sample (ΔΔCt = ΔCt_treated_−ΔCt_control_). The fold change in gene expression was calculated as 2^−ΔΔCt^. All experiments were conducted with at least three biological replicates, and data are presented as mean fold change ± standard error of the mean (SEM). Statistical significance was determined using a two‐tailed Student's t‐test, with a p‐value < 0.05 considered significant.

### Small RNA‐sequencing of EVs

EVs were isolated from 2D and 3D cultures of bone marrow‐derived MSCs and corneal‐derived MSCs. Total RNA, including small RNAs, was extracted using the Qiagen exoRNeasy Serum/Plasma Midi Kit, following the manufacturer's protocol. Three biological replicates were included for each group. RNA concentration and integrity were assessed using a NanoDrop spectrophotometer and an Agilent Bioanalyzer 2100. Based on Novogene's recommendations for EV samples, more than 10 ng of total RNA per sample was sufficient for library construction. Library preparation and sequencing were conducted by Novogene Co., Ltd. (Beijing, China) using the NEBNext Multiplex Small RNA Library Prep Set for Illumina. After adapter ligation, reverse transcription, and PCR amplification, the libraries were size‐selected to enrich for small RNAs, purified, and sequenced on an Illumina NovaSeq 6000 platform. The expression of known and novel small RNAs was quantified and normalized using the transcripts per million (TPM) method to account for differences in library size and sequencing depth. Differential statistics for 3D versus 2D were first computed separately for bone marrow and cornea samples using edgeR.^[^ ^90^, ^91^ ^]^ miRNAs with average counts <5 were removed from the dataset and assessed separately for each tissue. For bone marrow samples, we used the exactTest function. For cornea samples, generalized linear models (GLMs) were used to adjust for subject‐specific differences in a repeated measures model and to remove subject‐specific effects from the normalized expression using the removeBatchEffect function. In both tissues, TMM correction was performed to adjust normalized values for outlier genes, computed log‐scaled normalized expression was obtained using the cpm function, and adjusted p‐values for multiple testing using the false discovery rate (FDR) correction of Benjamini and Hochberg.^[^ ^92^ ^]^ The log‐scaled normalized expression levels for each tissue were merged, keeping only genes that passed the minimum expression filter in both. Differential statistics for bone marrow versus cornea were obtained using limma,^[^ ^93^ ^]^ and p‐values were again adjusted using the FDR correction. Annotated pathways and target genes for miRs were obtained using the microRNA target filter function in Ingenuity Pathway Analysis (IPA). Only high‐confidence predicted or experimentally observed targets were retained.

### Statistical Analysis

For downstream functional assays, we selected three independent EV preparations, each derived from a different donor randomly chosen from the original pool of five. All statistical analyses treated each donor‐derived EV preparation as a biological replicate. Technical replicates (e.g., repeated wells or measurements from the same donor EV batch) were averaged prior to statistical testing to avoid inflating sample size. All data in this study are reported as mean ± Standard Deviation (SD), with experiments conducted using EVs from five independent donors per MSC source. Each experiment included a sample size of 3 to 5 (n = 3–5) and was performed in biological triplicate unless stated otherwise. Normality was assessed using the Shapiro‐Wilk test. Based on the normality results, either a two‐tailed t‐test or a Mann‐Whitney test was performed using Prism software to evaluate statistically significant differences, with results presented as p‐values. Sample size for in vivo studies was determined a priori using G*Power 3.1. For the primary corneal nerve outcome, assumptions were Cohen's d = 1.5, a two‐tailed two‐sample t test, α = 0.05, and power = 0.80, yielding n = 4 per group. For von Frey, we modeled a repeated‐measures design (within–between interaction) with correlation among repeated measures = 0.5 and non‐sphericity correction = 1.0, α = 0.05, power = 0.80. Technical replicates were averaged prior to analysis. Randomization and outcome assessment were performed under blind conditions.

## Conflict of Interest

The authors declare no conflict of interest.

## Data Availability

The data that support the findings of this study are available from the corresponding author upon reasonable request.
